# The implications of regional and national demographic projections for future GMS costs in Ireland through to 2026

**DOI:** 10.1186/1472-6963-14-477

**Published:** 2014-10-21

**Authors:** Aisling Conway, Martin Kenneally, Noel Woods, Andreas Thummel, Marie Ryan

**Affiliations:** Department of Management & Enterprise, Cork Institute of Technology, Rossa Avenue, Bishopstown, Cork City, Ireland; Centre for Policy Studies, University College Cork, 6 Bloomfield Terrace, Western Road, Cork, Ireland; Darmstadt University, Karolinenplatz 5, 64289 Darmstadt, Germany

**Keywords:** GMS prescribing, Monte carlo simulation model, Regional variation, Scenario analysis

## Abstract

**Background:**

As the health services in Ireland have become more resource-constrained, pressure has increased to reduce public spending on community drug schemes such as General Medical Services (GMS) drug prescribing and to understand current and future trends in prescribing. The GMS scheme covers approximately 37% of the Irish population in 2011 and entitles them, inter alia, to free prescription drugs and appliances. This paper projects the effects of future changes in population, coverage, claims rates and average claims cost on GMS costs in Ireland.

**Methods:**

Data on GMS coverage, claims rates and average cost per claim are drawn from the Primary Care Reimbursement Service (PCRS) and combined with Central Statistics Office (CSO) (Regional and National Population Projections through to 2026). A Monte Carlo Model is used to simulate the effects of demographic change (by region, age, gender, coverage, claims rates and average claims cost) will have on GMS prescribing costs in 2016, 2021 and 2026 under different scenarios.

**Results:**

The Population of Ireland is projected to grow by 32% between 2007 and 2026 and by 96% for the over 70s. The Eastern region is estimated to grow by 3% over the lifetime of the projections at the expense of most other regions. The Monte Carlo simulations project that females will be a bigger driver of GMS costs than males. Midlands region will be the most expensive of the eight old health board regions. Those aged 70 and over and children under 11 will be significant drivers of GMS costs with the impending demographic changes. Overall GMS medicines costs are projected to rise to €1.9bn by 2026.

**Conclusions:**

Ireland’s population will experience rapid growth over the next decade. Population growth coupled with an aging population will result in an increase in coverage rates, thus the projected increase in overall prescribing costs. Our projections and simulations map the likely evolution of GMS cost, given existing policies and demographic trends. These costs can be contained by government policy initiatives.

## Background

Many of the developed economies, including Ireland, are struggling to contain expenditure on health following the fiscal crisis in the aftermath of the 2008 global economic recession. Expenditure on pharmaceuticals is one of the fastest growing elements of total health spending within the European Union (EU). Pharmaceutical expenditure exceeded €180 billion in 2008 within the EU and accounted, on average, for approximately 17 per cent of EU countries’ total expenditure on health [1]. In 2009, Ireland had one of the highest pharmaceutical spends per capita of the Organisation for Economic Co-operation and Development (OECD) countries after the US, Canada and Greece [2]. In Ireland, pharmaceutical expenditure accounted for 16.9 per cent of total health expenditure in 2007 and pharmaceutical spend per capita was €446.37, peaking at 17.9 per cent and €501.48 in 2010 [3]. Community Drugs Schemes (CDS) in Ireland consist of the General Medical Services (GMS), the Long Term Illness (LTI) Scheme, Drug Payment Scheme (DPS) and the High Tech Drug Scheme (HTDS). CDS expenditure on medicines accounted for approximately 85 per cent of total drug expenditure in Ireland in 2007 [4]. The annual cost of medicines under community drug schemes in Ireland increased from €564 m in 2000 to €1,961 m in 2009 before falling by an estimated 8 per cent by 2011 following a series of cost containment measures [1].

The focus of this paper is the GMS scheme which is the largest of the schemes with 36.9 per cent of the population eligible in 2011 [5]. Those who are eligible for the GMS scheme (medical card holders) are entitled to free prescription drugs and appliances with a nominal charge per item (€0.50) introduced in budget 2011 . Cost of Medicines on the GMS scheme more than trebled between 2000 and 2009 from approximately €338 million to €1,260 million. Overall, costs of medicines were approximately €1,048 million in 2007 and increased by 15.2 per cent to approximately €1,207 million in 2011.

Following a sharp contraction in the Irish economy in 2008, resulting in mounting fiscal deficits, the sustainability of funding these community drug schemes have come under the spotlight of the EU, International Monetary Fund (IMF) and European Central Bank (ECB) (Troika) who have set stringent targets for reducing drug costs as conditions of the bailout programme. Whilst the troika’s main cost containment measure is on the substitution from proprietary to generic prescribing, the challenge for Ireland and many of our EU counterparts in containing expenditure on pharmaceuticals is severely restricted by the growth in the elderly component of our populations, the sub-group with the greatest consumption of prescribed drugs. Projections by the Central Statistics Office (CSO) show the number of elderly people in Ireland will have grown by 200,000 by 2021 [6].

The literature highlights six cost drivers of drug expenditure; population growth, population aging, general inflation, price effects, volume effects and mix of drugs [7]. Whilst Irish drug pricing is substantially higher than our UK counterparts [8], the ESRI identifies population growth and population aging as the key drivers of future drug costs [2]. People in developed economies are living longer, with life expectancy at their highest level and population projections predicting significant increases in the total number of older people. The proportion of people who are very old is growing fastest and this number is expected to almost double by 2030. According to the World Health Organisation, the present and future generations of older people can also expect to live for considerably longer than their predecessors.

This paper assesses the implications of demographic change and policy scenarios on future GMS costs in Ireland from 2007 through to 2026. GMS costs are determined by population, GMS coverage, claims rate and the average cost per claim and assumptions were made around these four variables For this analysis, the coverage rate is defined as the proportion of the population eligible for the GMS scheme and the claims rate is the percentage of those covered who make a claim. The average cost per claim is the total cost of claimants divided by the number of claimants.

Predictions of drug utilisation and costs have been undertaken in several international studies [9, 10]. A fixed effects model was used to predict the impact of regulation on pharmaceutical cost in 19 countries from 1992 to 2004, finding that regulations reduce pharmaceutical revenues significantly [9]. A Spanish study investigated the capacity to predict future high-cost patients in Spain through c-statistic, sensitivity and specificity parameters finding that pharmacy-based predictive models can assist administrators and medical directors in planning the health budget and identifying high-cost-risk patients amenable to care management programs [10]. A number of predictive studies have been undertaken in the United States on future health care costs, which also inform future prescribing costs [11–13]. These studies predicted drug expenditures for the Medicare scheme [11] and a veteran health population [12], respectively, and their models included demographic and health status variables. Both [11, 12] found combined drug and diagnostic data are superior in predicting total health care costs. A comparison of the predictive performance of diagnosis and drug based models to determine health care costs in the US found drug based models predict future pharmacy costs more effectively than diagnosis based models and a combined drug and diagnostic model is a better predictor of future health care costs than either model alone [13].

In terms of the most relevant studies in Ireland, a linear current use model on 2006 data, and a regression model, was used to project future prescribing for the three community drug schemes [14]. They predicted that ingredient costs are likely to be between €1.5bn and €2.3bn for the three Community drug schemes (GMS, LTI & DPS) by 2020 with the largest increase in the GMS scheme. The ingredient cost incorporates approximately 80 per cent of the total cost of prescriptions. The total cost of prescriptions is comprised of the ingredient cost, dispensing fee and VAT. This paper updates and expands the Bennett et al. (2009) projections for the GMS scheme only. In addition, this paper includes demographic and regional population changes, policy variables and provides a Monte Carlo sensitivity analysis of the results. A regional examination of chronic conditions influencing drug prescribing found that regional factors were highly variable in Ireland and significant [15], whilst an investigation into the prescribing prevalence of insulin dependent and non-insulin dependent diabetes again found a significant variation between regions [16].

## Methods

The main source of data is the PCRS Statistical Analysis of Claims and Payments, Annual Reports and a sample 2007 PCRS database. The sample database consists of 192,000 observations extracted from the GMS population claims data. The base year used is 2007 and the geographical area is the former Health Board, of which there are 8. The first 100 observations by gender and age cohort in each monthly file was used in this analysis. For example, 0–5 Eastern male, there are 100 observations. 192,000/100 = 16,000 in each month. The variables in the database include; ingredient cost, VAT, dispensing fee, total cost, number of items and number of forms and are disaggregated by month (12), age cohort (10), gender (2) and region (8). Claims rate and average cost per claim were estimated using the sample PCRS database. For our analysis, the data is disaggregated by 10 age cohorts, 2 genders and 8 regions for each of the four variables. The data used in this paper was granted by the Health Service Executive (HSE) and is anoymised where individuals are not identified, the data does not have a unique user identifier. Therefore, it was unnecessary to seek ethical approval.

Population data was sourced from the CSO Regional Population Projections 2011–2026. Data was detailed by year of age, gender, county and region, taking account of international migration, fertility and inter-regional flows. In order to conform to HSE age cohorts, the CSO data were reconciled into ten age cohorts. The CSO regions (Border, Mid-East, Dublin) were not reconcilable with three of the old health board regions (North-West, North-East, Eastern). An adjustment factor based on the 2006 Census was applied to form the North-West, North-East and Eastern regions to conform to the old health board regions.

Coverage 2007 was estimated from the PCRS annual report and CSO population projections. Medical card coverage was 30.1 per cent (1,276,178 persons) of the population in 2007 [17] increasing to 36.9 per cent (1,694,063) in 2011 [5]. The minimum, mean and maximum 2007 scenarios were calculated based on historical coverage data between 1996 and 2010. Three scenarios were estimated for 2011 based on historical coverage data between 1996 and 2011 to form three coverage scenarios by region, gender and age cohort (RGA).

Coverage projections for 2016, 2021 and 2026 were estimated using 2011 coverage data, as this was the most up to date data available at the time of projections. The projected mean population was used to estimate the coverage and the number of eligible persons for each scenario by RGA in 2016, 2021 and 2026. If the older age cohorts (70–74, >75) for each region and gender exceeded 1.00, an adjustment factor was applied with lambda taking any value between 0 and 0.99. For example, if the projected population for >75 in a given region is 100 but the number of projected GMS eligible persons is 105, GMS coverage rate is 1.05. As this value exceeds 1.00, the adjustment factor below was applied to scale the value below 1.00.

The claims rate was estimated using both the PCRS 2007 annual report and a sample 2007 PCRS database. For this analysis, the claims rate is the proportion of medical card holders who make a claim. Of the 1,276,178 medical card holders in 2007, 1,225,131 made a claim on the card giving a claims rate of 96 per cent in 2007. A 95 per cent confidence interval was applied, establishing a lower bound (minimum scenario) and upper bound (maximum scenario) around the mean claims rate. For this analysis, the claims rate was assumed to remain at the 2007 level throughout the lifetime of the projections . This is borne out by It was found that the introduction of a 50 cent co-payment charge in 2010 did not affect patient demand for drugs [18]. Furthermore, the national claims rate has not changed since the co-payment introduction. 93 per cent of eligible GMS persons availed of services in 2010 and 2011 [5, 19].

The average cost (ac) per claim was determined from the sample 2007 PCRS database which is the total cost of claimants divided by the number of claimants. More than 96 per cent of all GMS eligible persons availed of services in 2007 with an average pharmacy cost of €856.14 per person [17]. A 95 per cent confidence interval was applied, establishing a lower bound (minimum scenario) and upper bound (maximum scenario) around the mean cost per claim. Historical ac per claim growth rates were determined and we assumed the historical growth evolution would continue in estimating the ac per claim for 2011 and projections between 2016 and 2026.

The utilisation and expenditure on drugs are difficult to forecast due to uncertainties about the rate of adoption of new medicines and various ongoing health care reforms and activities to improve the quality and efficiency of prescribing. The Monte Carlo Simulation (MCS) model employs statistical sampling to forecast under uncertainty. MCS generates a large number (100,000) of outcomes which is representative of your decision and assesses your decisions and the impact of risk, allowing for better decision making under uncertainty. Taking a large number of simulations as we have done, gives an excellent approximation to the true distribution of projected cost. The equation used for simulations is;

where error is an ensemble for the Monte Carlo simulation, having a normal distribution with zero mean and standard deviation of 5% of cost. It was found that using an MCS model avoided bias in health economic modelling [20]. The MCS model identifies uncertain variables (explanatory variables) and uncertain functions (dependent variables). The uncertain variables are population, coverage, claims rate and ac per claim and the uncertain function is the total costs of GMS scheme. For this analysis, the MCS model was adopted to forecast GMS costs through to 2026.

Empirical probability (proportion) was determined by each RGA class. The cumulative probabilities were calculated which gives a weight for the occurrence of finding a person in an RGA class. A macro was written in Visual Basic Editor in Microsoft Excel to run the simulations. An MCS with 100,000 iterations was used to propagate the uncertainty in the model. It’s important to assign an appropriate probability distribution to uncertain variables in developing a comprehensive MCS model [21]. A Normal distribution was applied to estimate costs to allow for uncertainty in the model. The MCS results were imported into Minitab 16 for statistical analysis. Descriptive and statistical analysis was performed in Minitab for each scenario and an overall analysis was carried out comparing all three scenarios by region, gender and age cohort. Statistical analysis included a histogram of cost, main effects plot, interval plot of cost by region, gender and age cohort.

## Results

The population of Ireland is projected to grow rapidly with a sharp increase in both the birth rate and in the elderly population. The total population is projected to grow by 96.3 per cent over the lifetime of the projections. The projections indicate that the population will increase from 4.3 million in 2007 to 5.7 million in 2026 (32.6% increase). The male population is estimated to increase by 33.4 per cent increase and the female population is estimated to increase by 30.2 per cent by 2026. In the 70 to 74 age category, there is an estimated 85.4 per cent increase between 2007 and 2026. The over 75 s shows the largest increase over the lifetime of the projections where the population more than doubles from 208,756 in 2007 to 422,589 in 2026 (102.4%). Over 70s projected population will increase in the first three time periods (2007–2011, 2011–2016 and 2016–2021) and will grow at a slower rate of 22.6 per cent between 2021 and 2026.

In terms of regional population change, the Eastern region constituted 35.5 per cent of the overall population in 2007. That proportion is projected to increase to 38.2 per cent by 2026 whereas the Southern region will decrease from 14.6 per cent in 2007 to 13.6 per cent in 2026. The remaining regions will see a decrease in population apart from the Western region which will remain constant.

**Figure 1:**
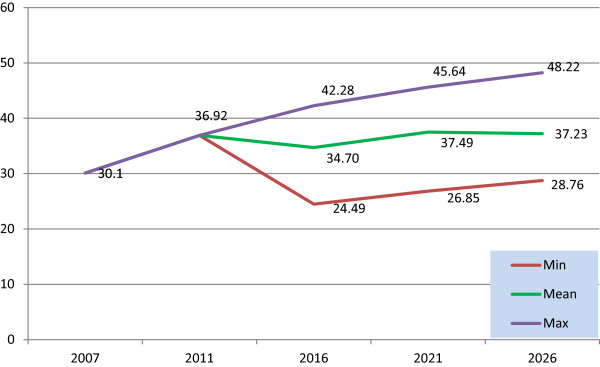
**Percentage GMS coverage rate projections, Ireland 2016–2026.**

We have estimated that coverage will be approximately 34.7 per cent in 2016, 37.5 per cent in 2021 and 37.2 per cent in 2026 (Figure 1). It is estimated that the number of eligible GMS persons will be approximately 1.8 million in 2016, 2 million in 2021 and 2.1 million in 2026. The estimates of the mean claims rate and the ac per claim for 2007 are detailed in Tables 1 and 2.

**Table 1:** **Mean scenario claims rate (Claims rate = Number of claimants/GMS population) 2007**

Region	0 – 11	12 – 15	16 – 24	25 – 34	35 – 44	45 – 54	55 – 64	65 – 69	70 – 74	>75	Total
**Eastern**											
Males	1.00	1.00	1.00	1.00	1.00	1.00	1.00	1.00	1.00	1.00	**1.00**
Females	1.00	1.00	1.00	1.00	1.00	1.00	1.00	1.00	1.00	1.00	**1.00**
**Midland**											
Males	1.00	0.99	0.99	0.99	1.00	1.00	1.00	1.00	1.00	1.00	**1.00**
Females	1.00	0.99	1.00	1.00	1.00	1.00	1.00	1.00	1.00	1.00	**1.00**
**Mid-Western**											
Males	0.93	0.88	0.90	0.91	0.91	0.94	0.96	0.9	0.97	0.99	**0.93**
Females	0.93	0.88	0.93	0.93	0.93	0.95	0.96	0.98	0.98	0.98	**0.95**
**North-Eastern**											
Males	0.88	0.82	0.85	0.86	0.87	0.91	0.93	0.95	0.94	0.95	**0.90**
Females	0.88	0.82	0.91	0.90	0.90	0.92	0.95	0.94	0.96	0.95	**0.91**
**North-Western**											
Males	0.87	0.81	0.83	0.83	0.84	0.88	0.91	0.93	0.95	0.94	**0.88**
Females	0.85	0.81	0.87	0.88	0.88	0.90	0.92	0.94	0.95	0.94	**0.90**
**South-Eastern**											
Males	0.96	0.94	0.94	0.95	0.96	0.97	0.98	0.98	0.98	0.99	**0.96**
Females	0.97	0.94	0.96	0.97	0.97	0.97	0.98	0.99	0.99	0.99	**0.97**
**Southern**											
Males	0.97	0.95	0.96	0.96	0.97	0.98	0.98	0.99	0.99	0.99	**0.97**
Females	0.97	0.95	0.97	0.97	0.98	0.98	0.99	0.99	0.99	0.99	**0.98**
**Western**											
Males	0.87	0.81	0.83	0.85	0.87	0.89	0.92	0.93	0.94	0.95	**0.88**
Females	0.87	0.81	0.89	0.88	0.89	0.91	0.93	0.94	0.95	0.95	**0.90**
**Total**	**0.93**	**0.90**	**0.93**	**0.93**	**0.93**	**0.95**	**0.96**	**0.97**	**0.97**	**0.98**	**0.95**

**Table 2:** **Mean scenario average cost per claim (€) by gender, age & region 2007**

Region	0 – 11	12 – 15	16 – 24	25 – 34	35 – 44	45 – 54	55 – 64	65 – 69	70 – 74	>75	Total
**Eastern**											
Males	228.09	352.31	468.36	663.13	715.28	1024.07	1225.56	1301.78	1111.03	1261.80	**835.14**
Females	250.13	314.96	370.39	434.52	622.62	1036.64	1182.49	1339.82	1233.99	1565.85	**835.14**
**Midlands**											
Males	252.32	346.52	570.72	862.57	843.18	1053.12	1212.19	1306.25	1374.38	1483.05	**930.43**
Females	286.53	287.65	433.68	527.21	784.65	1039.87	1280.93	1344.24	1510.02	1809.51	**930.43**
**Mid-Western**											
Males	312.55	518.26	632.72	780.04	943.63	946.39	1122.24	1150.38	1145.96	1224.23	**877.64**
Females	331.60	391.68	381.46	574.27	779.34	1001.62	1261.18	1233.93	1298.71	1522.60	**877.64**
**North-Eastern**											
Males	287.17	387.18	546.40	807.66	811.09	919.99	1136.46	1141.29	1192.14	1363.01	**859.24**
Females	290.24	438.25′	324.13	502.04	671.81	970.68	1129.80	1329.20	1337.70	1598.56	**859.24**
**North-Western**											
Males	224.88	313.54	411.39	607.13	721.31	792.40	909.40	976.33	958.41	1216.81	**713.16**
Females	257.50	264.09	294.35	388.02	566.78	767.27	1016.47	1076.26	1102.82	1398.05	**716.16**
**South-Eastern**											
Males	272.48	397.70	591.81	817.17	846.95	1009.93	1087.12	1150.66	1200.19	1286.47	**866.05**
Females	247.29	389.64	380.57	454.17	753.83	1114.55	1223.04	1277.73	1314.70	1504.99	**866.05**
**Southern**											
Males	310. 32	407.65	617.11	884.82	891.47	1039.17	1228.63	1315.09	1223.99	1265.83	**918.41**
Females	357.49	454.20	416.13	583.39	754.74	1132.37	1240.35	1375.76	1380.47	1489.19	**918.41**
**Western**											
Males	298.90	375.46	555.50	865.61	822.04	935.52	1069.00	1199.25	1124.97	1307.75	**855.40**
Females	321.00	322.29	339.24	563.25	744.94	1035.37	1123.41	1238.10	1330.83	1535.57	**855.40**
**Total**	**283.03**	**372.59**	**458.37**	**644.69**	**767.10**	**988.69**	**1153.02**	**1234.75**	**1240.02**	**1427.08**	**856.14**

**Figure 2:**
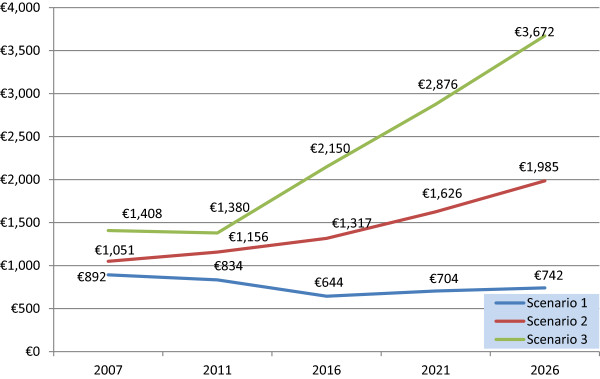
**Historical projections and projected pharmacy cost (€’000 m) to 2026.**

**Figure 3:**
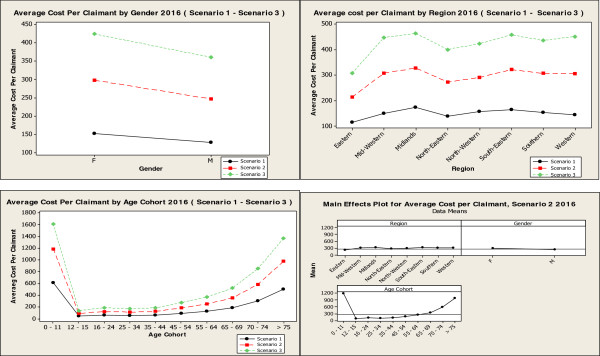
**Statistical analysis Scenario 1 – Scenario 3, 2016.**

**Figure 4:**
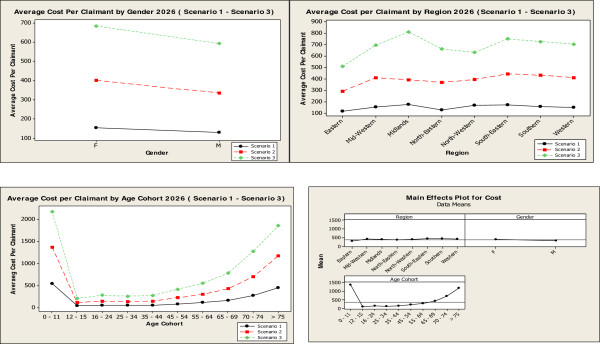
**Statistical analysis Scenario 1 – Scenario 3, 2026.**

The MCS model projects total pharmacy cost will rise to €1,317 million in 2016, €1,626 million in 2021 and €1,985 million in 2026 (Figure 2), given the assumptions around the 4 variables. The descriptive analysis of 100,000 simulations for scenario 2 in 2016 projected an average cost per claimant of €272.31. This is projected to be €312.61 in 2021 and €366.81 in 2026 (Table 3). Statistical analysis for the 3 scenarios in 2016 and the main effects plot for scenario 2, 2016 show the effect the three categorical variables have on the average cost per claimant in 2016. The main factors driving costs in 2016 were found to be: age, and in particular those aged 0–11, and those aged 70+, being resident in the midlands region and being female. These key findings were replicated in 2021 and 2026. The main effects plot illustrates age as the most significant driver of cost compared to the other two variables, gender and region (Figure 3). These results show a similar picture for 2021 and 2026 (Figure 4).

**Table 3:** **Descriptive analysis of average cost per claimant (€) 2016 – 2026 (Scenario 2)**

Year	# of Simulations	Mean	SE Mean	St. Dev	Min	Q1	Median	Q3	Max
**2016**	100,000	272.31	0.912	149.32	69.63	106.19	167.59	286.14	1541.72
**2021**	100,000	312.61	0.994	314.45	78.65	121.29	190.43	330.86	1579.73
**2026**	100,000	366.81	1.18	272.28	86.68	135.56	241.10	371.60	1602.28

## Discussion

Population growth, aging population and high fertility rates will be drivers of future health costs in Ireland. Population growth is the effect of changes in the size of the population on total drug spending. Other things being equal, an increase in population size will increase total drug spending. It is well documented in the literature that population is a driver of Irish health care expenditure [22, 23]. We estimate that the Irish population will have increased by approximately 33 per cent by 2026. Population aging is the effect of changes in the age distribution of the population on spending. An aging population will result in increased spending as the use and cost of drugs increase with age for the average individual within the population. Furthermore, Ireland has the highest fertility rate (2.05) in the European Union (EU27) as of March 2013. According to Eurostat, Ireland has on average two live births per woman [24]. The CSO recorded 72,225 births in 2012, a 19.3 per cent increase on 2002 births, indicating fertility will be a driver of population growth going forward [25]. This is compounded in our results with the 0–11 age cohort as a key driver of future health care costs. A Canadian study found population growth and population aging had the least effect on drug spending with volume effects and the mix of drugs having the most significant effect of the six cost drivers [7].

It is argued that a shift in the proportion of the population being elderly causes a shift in the health care expenditure in fifteen EU countries [26]. That is, as people are living longer, it represents a shift in expenditure from one age group to another. According to the latest CSO life tables, male life expectancy has increased by 5.8 years between 1996 and 2006 and 4.9 years for females [27]. This may justify the addition of a 12^th^ HSE age cohort, the >80s and renaming >75 s to 75–79 to represent a shift in population where people are living longer.

Population growth, aging population and high fertility rates under existing policies will have a knock on effect on future GMS coverage rates. That is, more people will be eligible for medical cards. Since July 2001, everyone over the age of 70 had a free entitlement to a medical card. In 2005, General Practitioner Visit Cards (GPVC) were introduced for persons who did not qualify for a medical card. This card allows one to obtain free general practitioner services subject to eligibility criteria. This initiative was extended to all children under five years of age in the Irish budget, 2014. In 2005, 5,079 people were entitled to a GPVC [28] but by 2011 this increased to 125,657 [5].

In January 2009, eligibility for the GMS scheme changed for the over 70s. New medical card income limits were introduced. A single person with an income of €700 per week (€36,500 per year) or a couple with an income of €1,400 per week (€73,000 per year) were no longer entitled to a free medical card [23]. Budget 2014 reduced the income limits to €500 per week for a single person and €900 per week for a married couple [29]. This policy change will reduce the eligibility rates and reduce overall coverage rates, holding all other factors constant. Since the introduction of this policy change, it is difficult to quantify the impact of this policy as the Irish economy is still in recession with more people eligible for the medical card due to their economic circumstances. Due to the three drivers of future health costs as mentioned above, GMS coverage is likely to increase thus increasing health costs. To counter this increase, the Irish government may need to consider further decreasing the income limits for medical cards or other measures.

The Midlands region is the most expensive and the North-West is the least expensive old health board region between 2003 and 2009 in terms of the average pharmacy cost per eligible GMS person. The Midlands cost ranged from €649.14 in 2003 to €943.28 in 2009 and the North-West ranged from €521.91 and €787.19 over the same time period [30]. Our results show that this trend is forecasted to continue to 2026. This regional variation in cost may be explained by the following two studies. The distribution of chronic conditions was examined across the old health board regions and it was found the Midlands had the highest prevalence of chronic conditions with the Western and North-Western regions having the lowest [15]. The prescribing prevalence of insulin dependent and non-insulin dependent diabetes was examined across regions [16]. They found the Midlands and the Mid-Western regions had the highest prescribing ratios for both types of diabetes and the North-Western region had the lowest prescribing ratio. It is well documented that females visit doctors more regularly than men, hence prescribing rates are higher for females with a resulting effect of higher health costs. Our results verify this, with females forecasting higher costs than males for all scenarios.

The claims rate, i.e. the proportion who make a claim on a medical card, has been above 90% since 2002. The Irish government introduced a patient co-payment system in October 2010 for medical card holders with a 50 cent per prescription charge, capped at €10 per month and in Budget 2014, this was increased to €2.50 per prescription item, with a monthly cap of €25 [29]. The prescription charge was introduced to reduce the claims rate and the average cost per claim. Research found the 50 cent co-payment charge did not affect patient demand for drugs [18]. No research has been done on subsequent increases in the patient co-payment Irish system. More expensive co-payment charges exist in the UK [31], Italy [32] and Australia [33]. These studies show that an expensive co-payment system can affect the utilisation and demand for drugs. It is well documented in the literature that co-payment charges can reduce the utilisation of both essential and non-essential medicines in vulnerable populations such as the poor, elderly and chronically ill [34, 35]. Therefore the Irish government needs to be mindful of the ill-effects of future increases in prescription charges as these groups comprise the GMS population in Ireland.

Limitations of Data: It is important to note the limitations of this research. The PCRS sample data lacked a unique user identifier. There is a possibility of some data appearing twice in the dataset as we can’t identify individual claims, but the influence of such cases is small given the size of the sample database (192,000). The old health board regions (8) are no longer comparable to the current HSE regional structure (4), which was changed in 2010. Further research with more recent data and cost projections using the current regional structure would prove very useful.

## Conclusions

Over the next decade, Ireland’s population will experience rapid growth. This growth coupled with an aging population will result in an increase in both the coverage rate and the claims rate, thus the projected increase in overall prescribing costs. However, these costs can be contained by government policy initiatives. A downward adjustment of the income eligibility limits can be used to curtail the coverage rates and upward adjustments to the co-payment patient prescription charges can be utilised to reduce the claims rate, thus curbing the overall average cost per claim. Age (youngest and oldest), females and the Midlands region were found to have the most significant effect on future drug costs in Ireland. These projections remain subject to substantial uncertainty given the variable nature of future economic trends and policy decisions.

## Abbreviations

GMS: General medical services
PCRS: Primary care reimbursement service
CSO: Central statistics office
EU: European union
OECD: Organisation for economic co-operation and development
CDS: Community drug scehemes
LTI: Long term illness
DPS: Drug payments scheme
HTDS: High tech drug scheme
IMF: International monetary fund
ECB: European central bank
HSE: Health service executive
RGA: Region, gender, age cohort
AC: Average cost
MCS: Monte Carlo simulation
GPVC: General practitioner visit card.
